# Graphene FETs with Low-Resistance Hybrid Contacts for Improved High Frequency Performance

**DOI:** 10.3390/nano6050086

**Published:** 2016-05-10

**Authors:** Chowdhury Al-Amin, Mustafa Karabiyik, Phani Kiran Vabbina, Raju Sinha, Nezih Pala

**Affiliations:** Department of Electrical and Computer Engineering, Florida International University, Miami, FL 33174, USA; mkara006@fiu.edu (M.K.); pvabb001@fiu.edu (P.K.V.); rsinh001@fiu.edu (R.S.); npala@fiu.edu (N.P.)

**Keywords:** graphene field-effect transistor (GFET), current-gain cut-off frequency, access resistance, capacitive coupling, radio frequency

## Abstract

This work proposes a novel geometry field effect transistor with graphene as a channel—graphene field-effect transistor (GFET), having a hybrid contact that consists of an ohmic source/drain and its extended part towards the gate, which is capacitively coupled to the channel. The ohmic contacts are used for direct current (DC) biasing, whereas their capacitive extension reduces access region length and provides the radio frequency (RF) signal a low impedance path. Minimization of the access region length, along with the paralleling of ohmic contact’s resistance and resistive part of capacitively coupled contact’s impedance, lower the overall source/drain resistance, which results in an increase in current gain cut-off frequency, *f_T_*. The DC and high-frequency characteristics of the two chosen conventional baseline GFETs, and their modified versions with proposed hybrid contacts, have been extensively studied, compared, and analyzed using numerical and analytical techniques.

## 1. Introduction

Graphene is a promising two-dimensional material exhibiting exceptionally high crystal and electronic qualities, which has fascinated researchers for decades. The richness of its electronic and optical properties include, but are not limited to, its high residual carrier concentration, mobility, Fermi velocity, high thermal conductivity, its perfect 2D body, optical transparency, and high mechanical stability [1,2,3,4,5], which have already revealed a cornucopia of potential engineering and application. Numerous research groups have seen graphene as a descendant of Silicon for analog devices, though the roadblock of graphene—its zero bandgap [6]—made it incapable of switching off field effect transistors (FETs) and, thus, inappropriate for logic devices.

An important metric for a radio frequency transistor’s performance measurement is its current gain cutoff frequency, *f_T_*. The cutoff frequency inversely depends on source/drain resistance, which is composed of contact resistance and access resistance, and their minimization ensures *f_T_* increment. The detrimental effect of access resistance on a graphene FET is much more prominent compared to that on other FETs. In addition to that, handing this access resistance in graphene field-effect transistors (GFETs) is much more challenging compared to other FETs. That is the reason why access resistance is getting a great deal of attention in GFETs, which prompted us to work on a viable way to reduce it. For example, the typical access resistance of silicon metal oxide field effect transistor (Si-MOSFET) is ~150 ohm-um [7], on the other hand, this quantity, for GFETs, is ~350 ohm-um and is 80% of the total device resistance [8]. In addition to this, in Si-MOSFET, the access region is highly doped by ion implantation in order to reduce access resistance [9], whereas, this type of high energy doping scheme is not viable for GFETs, where single or a few layer graphene form the device’s access region. Techniques to reduce GFET contact resistance have been proposed and reported by numerous groups [10,11,12,13,14,15]. Reduction of access resistance for enhanced performance in graphene FETs [16,17,18,19,20], in III-N high electron mobility transistors (HEMTs) [21], and in GaAs/AlGaAs HEMTs [22], have also been reported. In this work, we have proposed, studied, and extensively analyzed a GFET with hybrid contacts capable of simultaneously reducing the access resistance and contact resistance of the device. The capacitive coupled part of the contact reduces the contact resistance and provides a low resistance path for the high frequency signal. In addition, the extension towards the gate reduces the access region length and the associated resistance—the access resistance. The approaching capacitive extension towards the gate might introduce additional parasitic capacitance; however, the cumulative aiding effect of contact and access resistance reduction on high frequency performance is more significant and prominent than the detrimental effect of additional parasitic capacitances. The elimination of the access region by using a sophisticated fabrication method, e.g., a self-aligned process, could be a better way to handle access resistance; however, the proposed method offers a promising viable alternative, where complex/sophisticated lithographic techniques with smaller tolerances need to be avoided.

## 2. Theory

**Figure 1:**
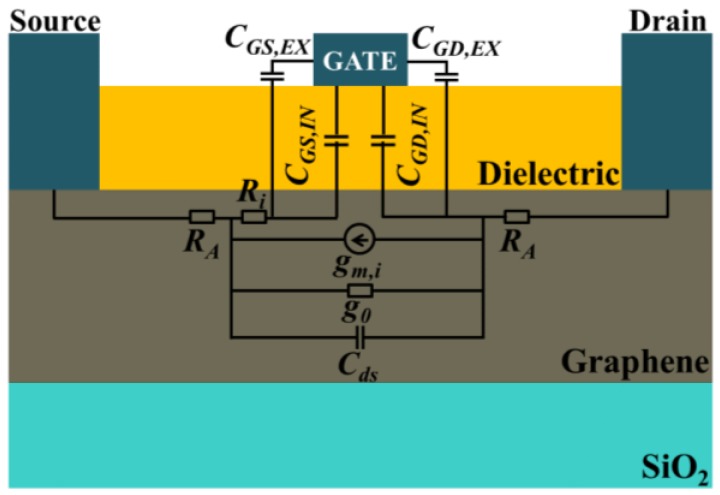
Small signal equivalent circuit overlaid on top of a conventional graphene field-effect transistors (graphene FET).

The small signal equivalent circuit of a conventional three-terminal GFET, overlaid on the device schematic, is shown in Figure 1.

The time that it takes the charge carriers to travel from the source to the drain is called the delay time, and can be divided into two parts: transit delay and parasitic delay. The intrinsic and extrinsic gate to source/drain capacitances are responsible for the transit delay and can be expressed as [23,24]:

(1) $$\tau_{TR}=\frac{\left(C_{GS,EX}+C_{GD,EX}\right)}{g_{m}}+\frac{\left(C_{GS,IN}+C_{GD,IN}\right)}{g_{m}}$$

On the other hand, parasitic resistances and capacitances cause a parasitic delay, as their names suggest, and can be expressed as [23]:

(2) $$\tau_{PAR}=[1+(1+C_{GS,PAR}/C_{GD,PAR})g_{0}/g_{m}]C_{GD,PAR}(R_{S}+R_{D})$$

where *g*_0_ = *1/R_SD_* is the output conductance, *R_SD_* is the drain to source resistance, *R_S_* and *R_D_* are the source and drain resistance consisting of ohmic contact resistance, *R_C_* and source/drain access resistance, *R_A_* in series:

(3) $$R_{D}=R_{S}=R_{C}+(L_{A}/\mu qn_{0}W)$$

where *L_A_* is the access region length (*L_GS_* and *L_GD_*), μ is the carrier mobility, *q* is electronic charge, *n*_0_ is the residual carrier density in graphene, and *W* is the device width. For simplicity, the effects of graphene doping, due to contacts and the gradient in carriers of the access region, have not been included; however, the effect is well explained in Reference [25]. In a common emitter configuration, the input terminal of a FET is the gate and the output terminal is the drain. As it is a FET, the input current in direct current (DC) is zero. As a result, the current gain for DC is theoretically infinite, h21 = i_out_/i_in_ = i_out_/0 = ∞. The reactance of gate to channel capacitance is inversely dependent on frequency, and with increasing frequency, the reactance decreases. As a result, the input alternating current (AC) current also increases with frequency, which results in a decrease of current gain. The frequency at which, current gain drops to unity is called the current gain cut-off frequency, and can be related to the total delay time in the device, as follows:

(4) $$1/2\pi f_{T}=\tau_{TR}+\tau_{PAR}$$

After substituting Equations (1) and (2) into Equation (4) and rearranging, the current gain cut-off frequency, *f_T_* of the GFET can be related to the small signal equivalent circuit parameters, as follows:

(5) $$f_{T}=\frac{g_{m}/\left(2\pi \right)}{[C_{GS}+C_{GD}]\times \left[1+(R_{S}+R_{D})/R_{SD}\right]+C_{GD}\times g_{m}\times \left(R_{S}+R_{D}\right)}$$

where, *R_SD_* is the total channel resistance.

**Figure 2:**
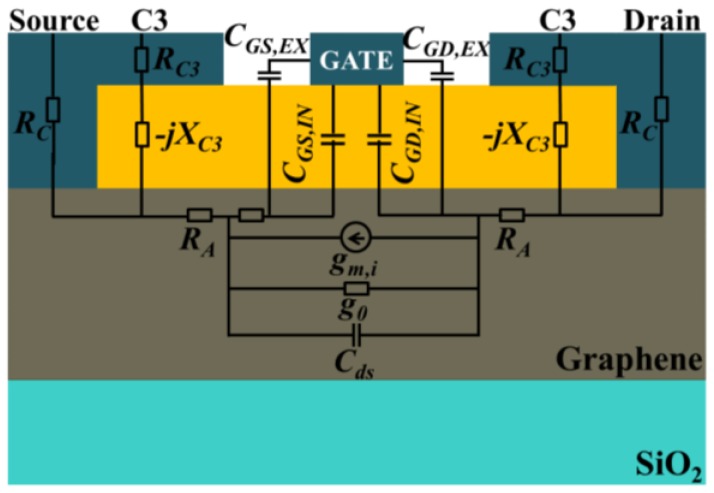
Small signal equivalent circuit overlaid on top of the proposed hybrid contact graphene FET.

If two capacitively coupled contacts (C3s) are placed on the access regions and connected to the ohmic source/drain, as shown in Figure 2, C3 will make a path for high frequency RF signal parallel to the ohmic contact. The C3 impedance, *Z_C3_* = *R_C3_*−*jX_C3_*, consists of real and imaginary parts, and the total contact impedance comes to be *Z_C_* = *R_C_*||*Z_C3_* = *R_C_*||*(R_C3_*−*jX_C3_)*. After rearrangement and simplification, *Z_C_* can be expressed as:

(6) $$Z_{C}=\frac{R_{C}^{2}R_{C3}+R_{C3}^{2}R_{C}+X_{C3}^{2}R_{C}}{{\left(R_{C}+R_{C3}\right)}^{2}+X_{C3}^{2}}-j\frac{X_{C3}R_{C}^{2}}{{\left(R_{C}+R_{C3}\right)}^{2}+X_{C3}^{2}}=R_{C}^{\prime }-jX_{C}^{\prime }$$

Assuming the length of C3 is *L_C3_*, the access region length of the hybrid contact GFET comes to be $L_{A}^{\prime }=L_{A}-L_{C3}$, and the new expression for source/drain impedance and total channel impedance becomes:

(7) $$\begin{array}{l} Z_{D}=Z_{S}=Z_{C}+R_{A}^{\prime }=R_{C}^{\prime }-jX_{C}^{\prime }+(L_{A}^{\prime }/\mu qn_{0}W)\\ Z_{SD}=2Z_{D}+R_{CH}=R_{SD}-jX_{SD} \end{array}$$

**Figure 3:**
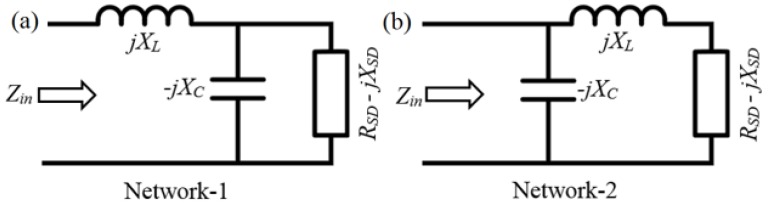
(**a**) Matching network-1; (**b**) matching network-2.

From the Equation (6), we can see that the total channel resistance has a real part, as well as an imaginary part. A simple matching network can be designed for matching and eliminating the imaginary part of the input impedance. An impedance matching network is an additional circuit that consists of a reactive element of such a value that can effectively nullify the opposite signed reactive element of the device, and thus eliminate the effective reactance of the whole system. It can be achieved with only two reactive elements that transform both the real and imaginary parts. A common two reactive element configuration is referred to as an L-section matching network, as shown in Figure 3a.

Considering network-1, we can quantify the input impedance as:

(8) $$Z_{in}=jX_{L}+\frac{R_{SD}+jX_{SD}}{1+jX_{C}R_{SD}-X_{C}X_{SD}}$$

To match it with a resistance, *R*, we consider *Z_in_* = *R*, and after equating the real and imaginary parts, we get:

(9) $$\begin{array}{l} X_{L}=\frac{1}{X_{CAP}}+\frac{X_{SD}R}{R_{SD}}-\frac{R}{X_{CAP}R_{SD}}\\ X_{CAP}=\frac{X_{SD}\pm \sqrt{\frac{R_{SD}}{R}}\sqrt{R_{SD}^{2}+X_{SD}^{2}-RR_{SD}}}{R_{SD}^{2}+X_{SD}^{2}} \end{array}$$

By solving these equations, we can determine the capacitor and inductor values required to nullify the imaginary part of the contact impedance. These two relations are derived for network-1 and are valid if *R_SD_* > *R*. On the other hand, if *R_SD_* < *R*, network-2, as shown in Figure 3b, needs to be used, and after following the same procedure, we can estimate *X_L_* and *X_CAP_* as follows:

(10) $$\begin{array}{l} X_{L}=\pm \sqrt{R_{SOURCE-DRAIN}(R-R_{SOURCE-DRAIN})}-X_{L}\\ X_{CAP}=\pm \frac{\sqrt{\left(R-R_{SOURCE-DRAIN}\right)}/R_{SOURCE-DRAIN}}{R} \end{array}$$

For devices working on a wide frequency range, a very common technique in RF/mobile communication, named the “frequency transformation technique”, needs to be used, as reported in Reference [26].

Once the matching network has been used, only the real part of contact resistance *R’_c_* remains, and the new expression of source/drain resistance comes out to be:

(11) $$R_{D}=R_{S}=R_{C}^{\prime }+(L_{A}^{\prime }/\mu qn_{0}W)$$

One can easily acquire the relation between *f_T_* and *Z_C_* by plugging this new *R_S_* and *R_D_* into the *f_T_* equation in Equation (5).

C3 can be considered as an RC transmission line and its impedance can be analytically calculated [27]. If a C3 is placed on top of the gate dielectric, the contact metal and graphene channel, with the in-between dielectric material, form an RC transmission line. The propagation constant, γ, and characteristics impedance, *Z*_0_, of this transmission line can be estimated by the following equations:

(12) $$\gamma =\sqrt{i2\pi R_{sh}C},Z_{0}=\frac{1}{W}\sqrt{\frac{R_{sh}}{i2\pi fC}}$$

where, *R_sh_* is sheet resistance of the graphene channel, *C* is the metal to graphene capacitance per unit area, *W* is the width, and *f* is the frequency. The C3 impedance can be estimated to be equal to the input impedance of this open-ended transmission line, as follows:

(13) $$Z_{in}=Z_{0}\text{coth}\left(\gamma L_{C3}\right)$$

**Figure 4:**
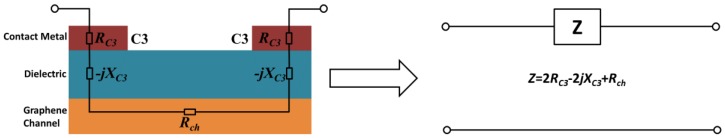
Schematic of a radio frequency (RF) transmission line method (TLM) structure on graphene, with a small-signal equivalent circuit overlaid on top, and the equivalent two-port network.

In simulations, the impedance of C3s can be calculated by using RF transmission line method (TLM) structures, with multiple C3s with various in-between distances. Two C3s and the graphene channel in-between is a two-port network, as shown in Figure 4, and its impedance can be estimated by extracting the two-port S-Parameters and converting them to a B-Parameter [28]. The real and imaginary parts of the B-parameter are actually the real and imaginary parts of total impedance of the two-port network—two C3 impedances, in addition to the in-between graphene channel resistance.

## 3. Results and Discussion

We started our analyses by simulating the DC and RF characteristics of a conventional graphene FET, reported in [29]. This was one of our baseline devices, and we named it GFET-1. The width of this device, as well as that of all other devices simulated in this work, was 100 µm.

**Figure 5:**
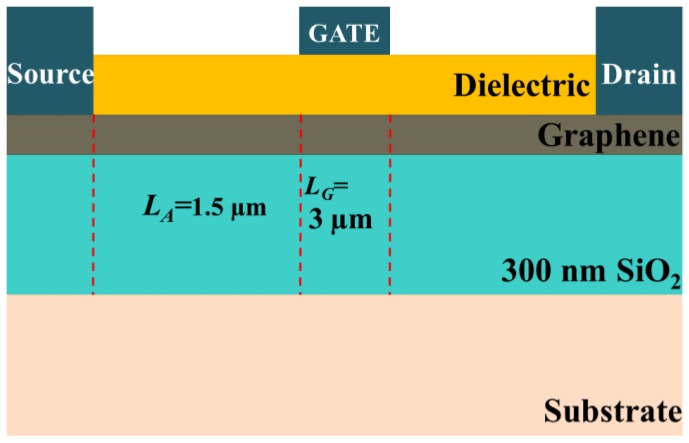
Schematic of baseline GFET-1 (not to scale).

The gate length of the baseline GFET-1 was 3 µm, gate dielectric thickness was 24 nm, and the access region length was 1.5 µm, as shown in Figure 5. Chemical vapor deposition (CVD) graphene with a sheet resistance of 210 Ω/□ and a hole (electron) mobility of 530 cm^2^/V·s (336 cm^2^/V·s) formed the device channel on 300 nm of SiO_2_.

**Figure 6:**
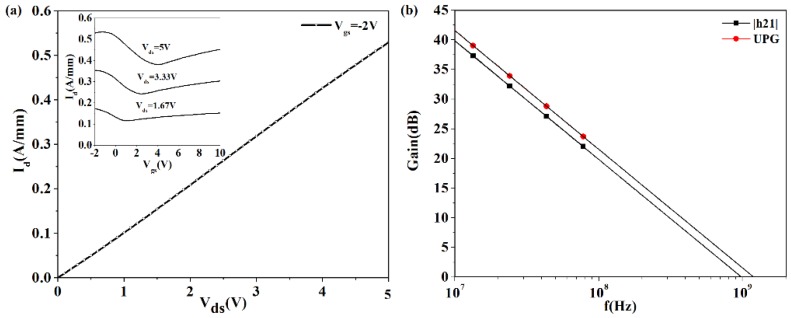
The I_d_-V_d_ characteristics and I_d_-V_g_ characteristics (inset) of the baseline GFET-1. RF characteristics (Current Gain, |h_21_| and Unilateral Power Gain, UPG) of the baseline GFET-1 plotted in decibel (dB) with respect to frequency.

We used a commercially available physically-based numerical technology computer aided design (TCAD) device simulation tool (Silvaco Atlas, Santa Clara, CA, USA) [30] and a modified material parameter for graphene to simulate and replicate the reported DC and RF characteristics of the baseline GFET-1. The tool solves electromagnetic and transport differential equations to calculate the electrical performance of a device modeled in DC, AC, or in transient modes of operation [31]. The simulated DC and high frequency characteristics of GFET-1 are shown in Figure 6. The simulated device characteristics are in a very good agreement with the reported ones [29], which also validates our method of simulation.

The sheet resistance of the graphene channel extracted from our simulation was 216 Ω/□. We aim to add two C3s to this device and short them to the ohmic contacts to extensively analyze their effects on the device’s high frequency performance.

**Figure 7:**
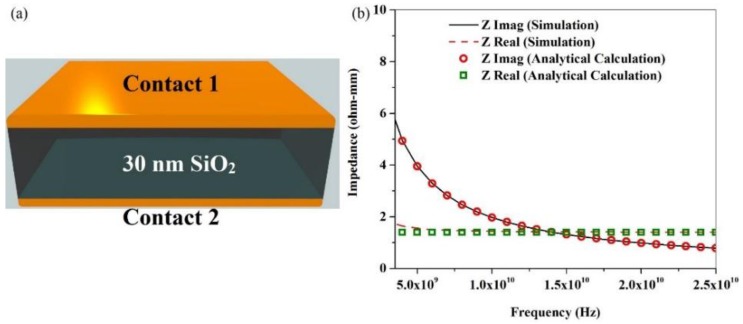
(**a**) Schematic of the capacitor like structure; (**b**) The real and imaginary parts of impedance, estimated from simulations and analytical calculations.

As a starting point of capacitive impedance simulation, we first simulated a simple capacitor-like structure. It consisted of 30 nm of SiO_2_ between two metal contacts, and each metal contact had a contact resistance of 0.7 ohm-mm, as shown in Figure 7a.

The real and imaginary parts of this capacitive impedance were estimated using simulations, as well as analytical techniques. In Figure 7b, the real and imaginary parts of the capacitive impedance estimated from simulation and analytical calculations are plotted with respect to frequency. We can see that the results using both methods are in a very good agreement, which validates our simulation technique of estimating capacitive impedance. For further verification, we successfully regenerated the experimental data for III-N RF TLM structures reported in Reference [27]. To estimate the impedance of the capacitance formed between a C3 and graphene channel with a gate dielectric in-between, we simulated an RF TLM structure on graphene having two C3s with various in-between distances.

**Figure 8:**
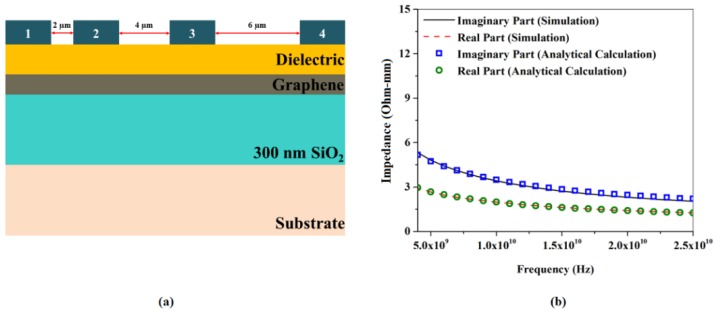
(**a**) Schematic of RF TLM structure on graphene; (**b**) the real and imaginary part of C3 impedance estimated from both simulation and analytical calculations, plotted with respect to frequency.

The C3s were placed on exactly the same structure as in the baseline GFET-1, consisting of 9 nm of SiO_2_ and 15 nm of Al_2_O_3_ serving as the gate dielectric, deposited on CVD graphene with a carrier mobility the same as that of baseline GFET-1, as shown in Figure 8a. The impedance between contact 1 and 2, 2 and 3, and 3 and 4 were calculated at a specific single frequency, plotted with respect to distance, and extrapolated up to zero distance to extract the real and imaginary parts of a single C3 impedance at that frequency. This procedure was repeated over the frequency range of 5 GHz to 25 GHz, with a step size of 1 GHz. The real and imaginary parts of C3 impedance, plotted with respect to frequency, are shown in Figure 8b.

**Figure 9:**
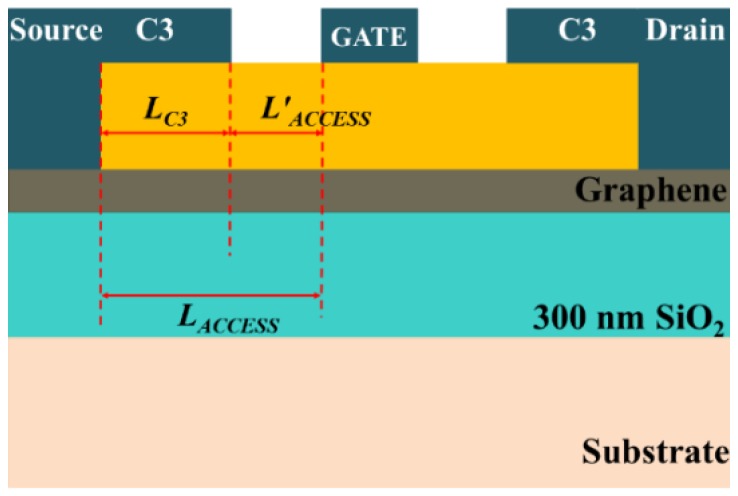
Schematic of the proposed GFET (not to scale).

**Figure 10:**
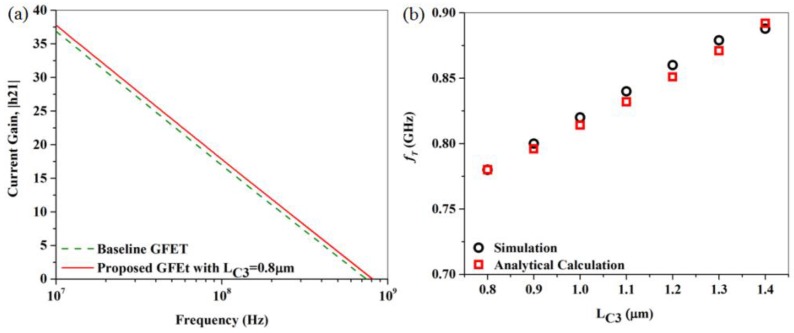
(**a**) Current Gain, |h21| of the baseline GFET-1 along with that of the proposed hybrid contact GFET in the electron regime (V_gs_ = +2.0 V and V_ds_ = +5.0 V) plotted with respect to frequency; (**b**) The current gain cut-off frequency (*f_T_*) of the proposed GFET extracted from |h21| *vs.* *f* characteristics, plotted with respect to *L_C3_*.

Finally, we simulated the proposed GFET, which has two C3s shorted to the ohmic source/drain contacts of the already simulated baseline GFET-1, as shown in Figure 9. The length of capacitively coupled extension was 0.8 µm in this simulation. The current gain, |h21| of the baseline GFET-1 and the proposed modified version with C3s are plotted with respect to frequency in Figure 10a. According to the definition, the frequency at which current gain becomes 0 dB is the current gain cut-off frequency, *f_T_*. We can see from Figure 10a that, for a C3 length of 0.8 µm, the *f_T_* of this proposed GFET reached a value of 0.78 GHz, whereas that of the baseline GFET-1 was 0.74 GHz. In each and every numerical calculation, the gate to source/drain parasitic capacitances have been considered. In addition to that, in analytical calculations, the parasitic capacitances have been estimated using geometric and material parameters. The value of these parasitic capacitances ranged from 3.90 × 10^−13^ F to 4.40 × 10^−13^ F.

The drain to source voltage, as well as the drain side C3 to source voltage, V_ds_, was 5.0 V during the frequency domain AC simulation. As the drain bias, as well as the drain side C3 bias, were positive, we considered the GFET electron regime operation so that the drain side C3 bias accumulated more major carriers (electrons) underneath. A gate bias of V_gs_ = 2 V was used to operate the GFET in the electron regime.

We later gradually increased the length of the C3s. The approaching C3 towards the gate reduced the access region length, as well as access resistance. Additionally, the increment of capacitive coupling area due to the C3 length increment decreased the capacitive impedance. As a result of access resistance decrease, as well as the decrease in capacitive impedance, the *f_T_* of the proposed GFET increased further. The effect of increased C3 length over *f_T_* for this device is shown in Figure 10b, estimated from both simulations and analytical calculations. As we can see from Figure 10b, the *f_T_* of this proposed device reached a value of 0.89 GHz for a C3 length of 1.4 μm, whereas it was just 0.78 GHz for a C3 length of 0.8 μm previously. Further incraese of C3 length was studied, and, due to introduction of high parasitic capacitance, it resulted in *f_T_* deterioration.

As the C3 impedance is dependent on frequency and from our results in Figure 6b, it was found that the real part of C3 impedance is reduced at higher frequencies; we intended to quantify the effect of C3 on RF performance for a shorter channel higher mobility GFETs. To do so, as before, a short channel high mobility GFET, reported in Reference [23], was chosen as our short channel high mobility baseline, and was named GFET-2. The device had a CVD-grown graphene channel with a carrier mobility of μ = 2234 cm^2^/V·s on a sapphire substrate with a gate length of 210 nm, and a source to drain distance of 1.5 μm. We considered the device geometry to be symmetrical and estimated the access region length to be 645 nm on each side of the gate. We simulated the DC and RF characteristics of the baseline GFET-2 as before, and they were in a very good agreement with the reported data [23]. For this simulation, as well as for the following simulations and analytical calculations, the device width was considered to be 100 μm, as before. Later, we simulated our proposed short channel high mobility GFET with a hybrid contact by making a capacitive extension of 245 nm of both the source and the drain towards the gate.

**Figure 11:**
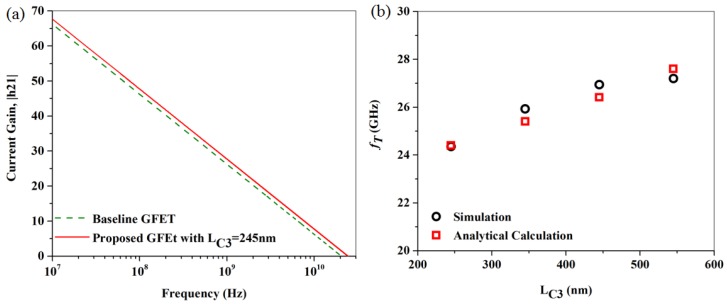
(**a**) Current Gain, |h21| of the baseline GFET-2 along with that of the proposed hybrid contact GFET in the electron regime (V_gs_ = +0.6 V and V_ds_ = +1.6 V) plotted with respect to frequency; (**b**) The current gain cut-off frequency (*f_T_*) of the proposed GFET extracted from |h21| *vs.* *f* characteristics, plotted with respect to *L_C3_*.

The RF characteristics of the baseline GFET-2 in the electron regime, along with that of the proposed GFET, having a C3 length of 245 nm, are shown in Figure 11a. From Figure 11a, we see that the *f_T_* of the baseline reported GFET and the proposed GFET with a 245-nm capacitive extension, are 20.05 GHz and 24.4 GHz, respectively. Later, the C3 length was gradually increase up to 550 nm as shown in Figure 8b. Due to the increase of the C3 length, the *f_T_* gradually increased and eventually reached a value of 25.9 GHz. As before, further increase of C3 length was studied, and *L_C3_* = 550 nm was found to be the optimum extension.

In addition to the C3 extension over the access region, we also simulated a GFET with the source to drain distance the same as that of the baseline, but with a longer gate. The new length of the gate was equal to old gate length plus 2*L_C3_*, *L_g-new_* = *L_g-old_* + 2*L_C3_*. From our simulations, we found that this device does not show any improvement of *f_T_*, rather the *f_T_* deteriorates compared to the baseline GFET. The reason behind this deterioration is the increase in transit delay. Though the C3 is capacitively coupled to the channel, as the gate contact is, the switching of the device takes place in the gate, not in the C3s. The increase of the gate length increased the transit delay, whereas the equal C3 extension length reduced the parasitic delay.

## 4. Conclusions

In this work, we have proposed and analyzed a novel geometry GFET with an ohmic source/drain and its capacitive extension towards a gate in order to overcome the set of limitations on its high-frequency performance that arises from contact resistance and access resistance. The extended part of the ohmic contacts over access region, not only reduces access region length and its corresponding access resistance, but also its capacitive coupling to the graphene channel provides a low resistance path for the high frequency signal. From our analyses, we found that our proposed long channel low mobility GFET with hybrid contacts has a current gain cutoff frequency that is 20% higher than the experimental data reported in the literature for the same geometry GFET with conventional ohmic contacts. On the other hand, the improvement for a short channel high mobility GFET with hybrid contacts was even more prominent, and had a current gain cutoff frequency 26.3% higher than that of the reported geometry conventional contact GFETs. The proposed devices would be easier to fabricate with a higher tolerance, and suitable for high frequency analog applications.
